# Signaling pathway networks in frozen shoulder fibrosis: from inflammatory initiation to fibrotic progression

**DOI:** 10.3389/fmed.2026.1883535

**Published:** 2026-08-07

**Authors:** Tianyu Guo, Yuhui Hua, Xin Liu, Bo Hu, Chenwen Wang, Jinbing He, Wei Zhou

**Affiliations:** Department of Orthopedics, Liyuan Hospital, Tongji Medical College, Huazhong University of Science and Technology, Wuhan, China

**Keywords:** fibrosis, frozen shoulder, molecular mechanisms, NF-κB, signaling, TGF-*β*

## Abstract

Frozen shoulder, also known as adhesive capsulitis, is characterized by fibrosis, thickening, and contracture of the shoulder joint capsule. Through a systematic review of existing research, we have analyzed the complex molecular network of shoulder fibrosis. Previous studies have found that the disease begins with inflammation of the nuclear factor kappa B (NF-κB) and leptin/Janus kinase/signal transducer and activator of transcription (JAK–STAT) pathways, and the transforming growth factor-beta (TGF-*β*) pathway is the key cause of fibrosis. Wingless/Int (WNT) and potentially Hippo pathways may contribute to fibrotic progression. The phosphatidylinositol 3-kinase (PI3K)/protein kinase B (PKB/Akt), mitogen-activated protein kinase (MAPK), and hypoxia-inducible factor (HIF-1) pathways are also related to abnormal cell proliferation, inflammatory response, and hypoxia adaptation, respectively. Technologies such as single-cell sequencing also show the heterogeneity of fibroblasts and their role in pathological networks. We point out the current limitations of pathway interaction research and look forward to the future use of multi-omics and spatial technology to gain an in-depth understanding of the mechanism and provide a theoretical basis for the formulation of treatment strategies for key nodes.

## 1 Introduction

### 1.1 Academic background

**Figure 1:**
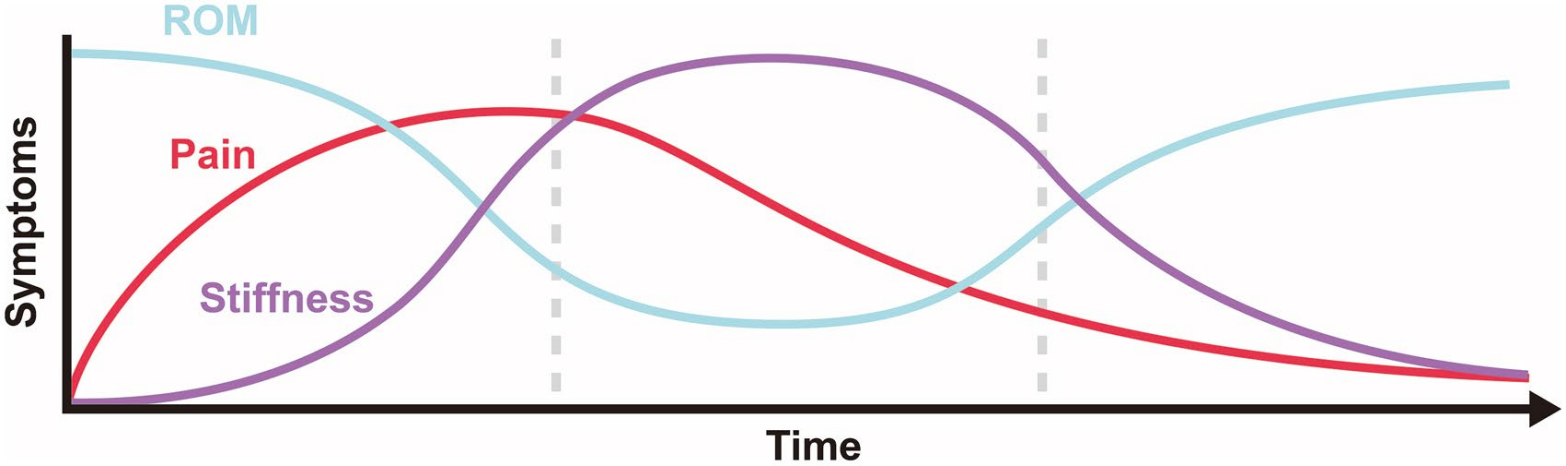
The natural history of frozen shoulder is divided into three stages. The freezing phase is characterized by progressively worsening pain, while the ROM is still relatively retained. The frozen period is characterized by stable pain with gradual stiffness, which seriously limits shoulder mobility and impairs daily functioning. The thawing period is defined as a spontaneous decrease in pain, and ROM gradually recovers over a long period of time.

Frozen shoulder is a common shoulder disease manifested by shoulder pain, stiffness, and limited range of motion (ROM). Generally speaking, this disease may go through three stages: freezing period (pain), frozen period (pain and stiffness), and thawing period (stiffness gradually relieved) (Figure 1). Pathologically, the inflammatory response of the joint capsule and its fibrosis are considered to be the basis of the disease (1). Fibrosis is considered to be the main mechanism underlying loss of shoulder function. This is mainly caused by abnormal fibroblasts in the capsule and excessive deposition of extracellular matrix (ECM) (thickening and contraction of the capsule wall; (2, 3)). Therefore, it is important to understand the cause of the development of fibrosis to understand the pathology of frozen shoulder.

Recent studies aim to figure out the molecular mechanism behind frozen shoulder fibrosis to find a new target. Now we know that its occurrence and development are the result of the complex interaction of genetic factors, metabolic disorders, abnormal immune function, and local inflammatory processes, and they are also accompanied by abnormal activity of a variety of cytokines, growth factors, and signaling pathways (1, 2, 4). Inflammation and fibrosis are factors closely related to frozen shoulders. Chronic inflammation can lead to the continuous release of pro-inflammatory and fibrotic factors, active proliferation of fibroblasts, excessive accumulation of ECM, and abnormal remodeling. These changes eventually cause pathological fibrosis and dysfunction of the joint capsule (2, 5). In general, systematic research and analysis of the signaling pathways related to the process of frozen shoulder fibrosis not only helps to clarify its pathogenesis but also provides a theoretical foundation for new targeted interventions.

### 1.2 Epidemiology

Previous studies have also pointed out that frozen shoulder is not an isolated local injury. Its development is closely related to a variety of systemic factors, such as metabolic and endocrine diseases. The first is the metabolic factor. Diabetes has been considered a risk factor for frozen shoulder. Existing studies show that the proportion of frozen shoulders in diabetics is close to 40%, compared with only 2–5% in the general population, and the prognosis of most patients is poor (6–10). Secondly, endocrine diseases, such as abnormal thyroid function, are also considered important risk factors (11, 12). Studies show that hyperglycemia, insulin resistance, and abnormal thyroid levels may cause chronic low-grade inflammation and metabolic disorders in the whole body, forming a network of metabolic inflammation. These changes may reduce the local injury repair threshold of the shoulder capsule tissue because even slight stimulation may trigger abnormal fibrosis repair, leading to disease (13–17). In addition to metabolic and endocrine factors, the development of frozen shoulders is also related to local biomechanical changes and neurological dysfunction (18). For example, abnormal pressure, movement pattern, or nerve loss on the joint may interact with the process of inflammation and fibrosis, leading to joint capsule contracture and functional impairment.

## 2 Pathophysiology and anatomical basis of frozen shoulder

**Figure 2:**
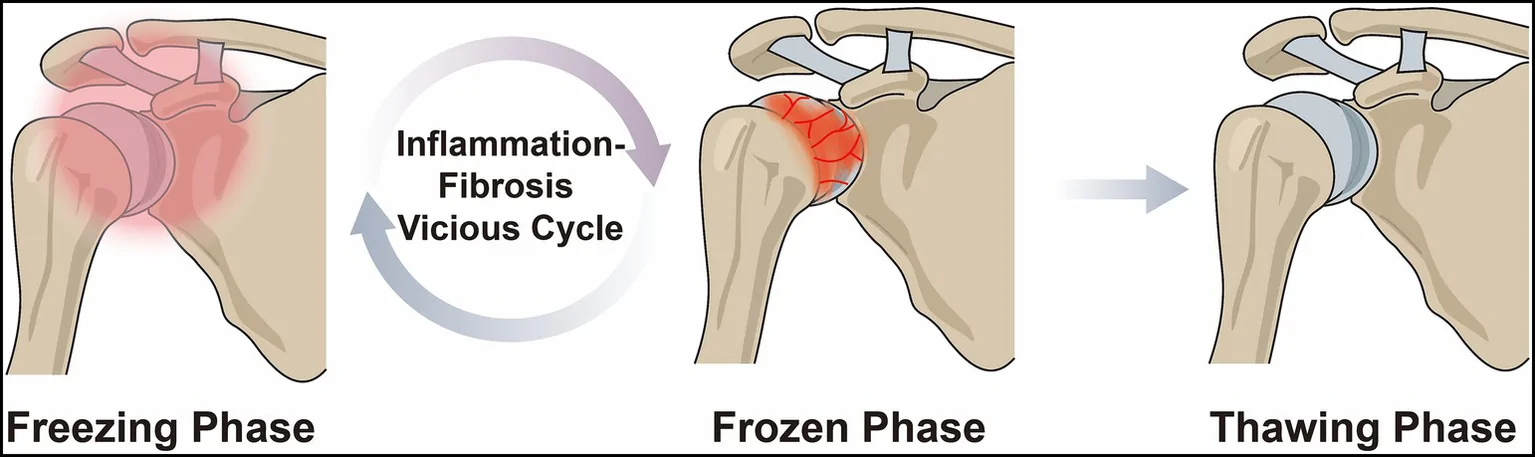
Schematic illustration of the clinical phases and pathophysiological mechanisms in adhesive capsulitis. In the freezing phase, synovitis and inflammatory mediator release predominate, with minimal structural alteration. In the frozen phase, abnormal fibroblast activation and proliferation, excessive collagen deposition, thickening of the coracohumeral ligament, and marked reduction of axillary recess volume produce capsular contracture and stiffness. In the thawing phase, inflammation subsides, fibrosis slowly resolves, and capsular volume partially recovers, allowing gradual improvement in range of motion.

The shoulder joint is the joint with the largest range of human movement. The soft tissue capsule of the joint is composed of the joint capsule and the ligament complex, which provides stability. The shoulder capsule is mainly composed of dense type I collagen and elastic fibers and contains blood vessels and nerve fibers. The most common cells in the joint capsule are fibroblasts. These cells maintain the stability and function of the joint capsule by synthesizing and secreting ECM proteins, providing static stability and flexible support for the joints (19). When the shoulder is frozen, the homeostatic state of the joint is destroyed. The main characteristics of this pathological destruction are the abnormal activation and abnormal proliferation of joint capsule fibroblasts, as well as collagen (20), in the matrix, accompanied by persistent inflammatory as well as the pathological formation of blood vessels and nerves (21–24). The resulting micropathological damage causes structural remodeling and functional impairment of the joint capsule. The abnormal collagen deposit reduces the volume of the entire joint capsule, thickens the capsule wall, and weakens the elasticity, resulting in fibrotic contracture and stiffness of the shoulder capsule. This structural change directly affects the ROM, which may eventually lead to severe restrictions on the shoulder joint during movement (Figure 2).

Clinical imaging and arthroscopic analysis also showed the core pathological area of frozen shoulder. At the anatomical level, pathological changes are mainly concentrated in the subscapular muscle, the deltoid muscle gap, and the bicipital humerus ligament (25–27). From the perspective of morphology and biomechanics, the pathological characteristics of this region are very obvious. The average thickness of the coracohumeral ligament (CHL) in patients with frozen shoulders increased to about 3.99 ± 1.68 mm, while in healthy controls, it was 3.08 ± 1.32 mm (28). Thickening and hardening will activate the relevant fibroblasts and abnormally deposit collagen in the local structure. The most typical is the sharp decrease in the volume of the axillary recess, which can be reduced from about 0.88 mL to 0.53 mL or less (8). This loss of volume, coupled with the contracture of the beak ligament, directly limits the mobility of the glenohumeral joint in the direction of rotation and extension, thus forming the direct anatomical basis for joint stiffness in frozen shoulder.

## 3 Inflammatory initiation phase: activation of NF-κB and leptin/JAK–STAT signaling pathways

The early process of frozen shoulder is mainly manifested as persistent inflammation, in which NF-κB and leptin/JAK–STAT signaling pathways play a key role in activation and regulation. Together, they create a molecular environment that promotes the transformation of joint capsule fibroblasts into inflammatory and fibrotic states. The NF-κB pathway is responsible for quickly starting local inflammatory reactions, while the leptin/JAK–STAT axis links metabolic abnormalities and local injuries of the whole body. They all play a role in the early inflammatory stage of the frozen shoulder and jointly promote the development of the disease to the stage of chronic fibrosis.

### 3.1 NF-κB signaling pathway

The NF-κB signaling pathway is the core hub for triggering the inflammatory response. This pathway can be activated by a variety of upstream signals, including inflammatory mediators, such as tumor necrosis factor-alpha (TNF-*α*), interleukin-1 (IL-1), and interleukin-6 (IL-6); oxidative stress; and cell damage. At the initial stage of inflammation of the frozen shoulder, its activation is very important. It directly regulates the expression of a variety of pro-inflammatory mediators and establishes an initial inflammatory microenvironment conducive to fibrosis (29, 30).

#### 3.1.1 Activation mechanism of the classical NF-κB signaling pathway

**Figure 3:**
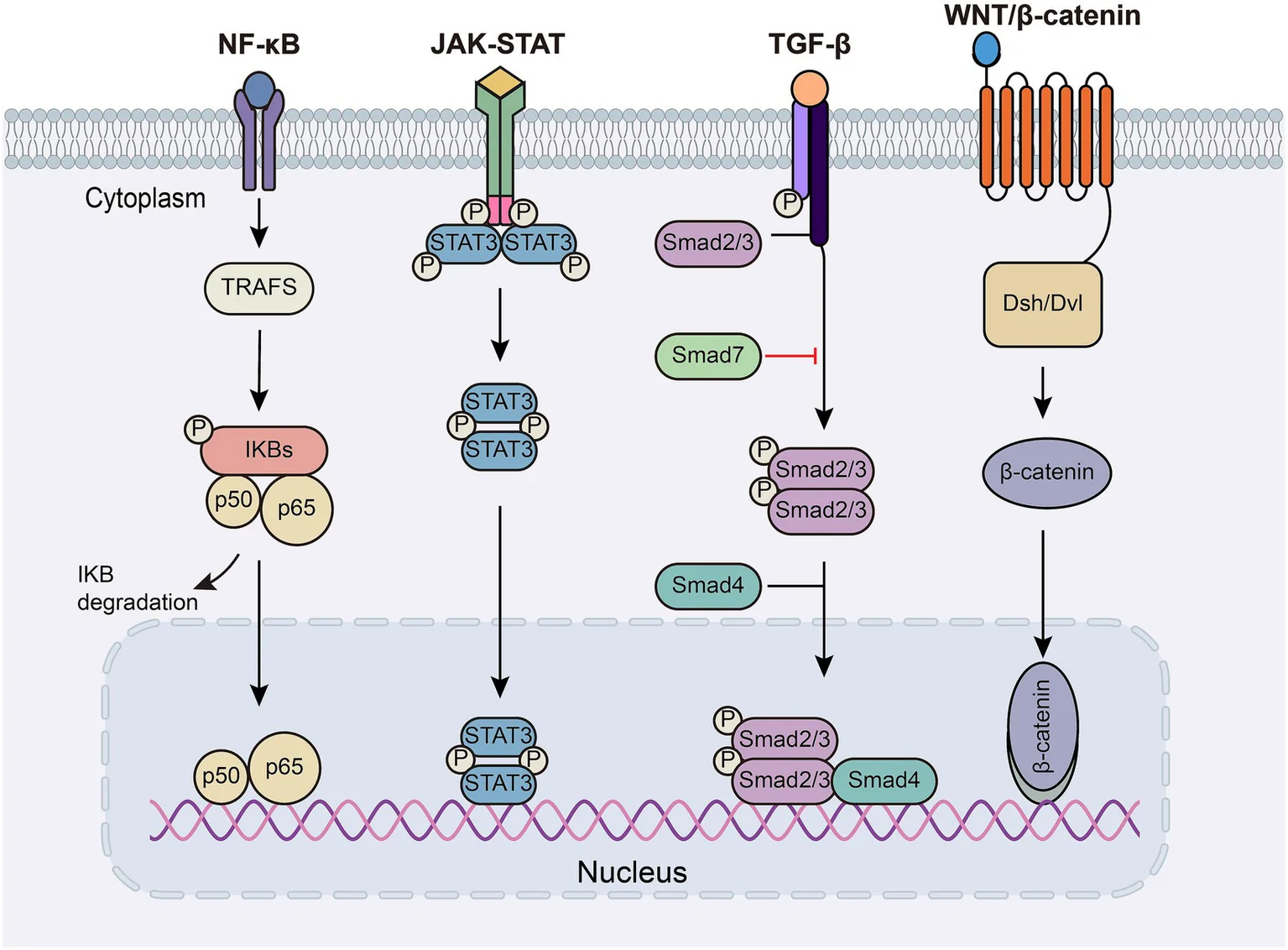
Schematic representation of the major signaling pathways implicated in the inflammatory initiation phase and the fibrotic formation phase of frozen shoulder.

The canonical NF-κB signaling pathway shows that in the resting state, NF-κB dimers, such as p50/p65, are bound to the cytoplasm by the IκB proteins. When cells are stimulated by upstream signals, Toll-like receptors (TLRs), tumor necrosis factor receptors (TNFRs), or interleukin-1 receptors (IL-1Rs) will dimerize and then recruit downstream signaling molecules, such as TNF receptor-associated factor 6 (TRAF6), through adaptor proteins. As an E3 ubiquitin ligase, TRAF6 catalyzes the formation of the ubiquitin chain of K63-linked, thus recruiting and activating the TAK1 complex. Subsequently, TAK1 is phosphorylated and activates the Ser/Thr-specific IκB kinases (IKKs). The activated IKKs will phosphorylate IκB so that it can be recognized and labeled by the E3 ubiquitin ligase *β*-Trcp, eventually causing it to be degraded by proteasomes. The degradation of IκB will release NF-κB dimers, which will be quickly transported to the nucleus (Figure 3). By binding to the promoter region of a specific gene, they regulate the transcription of a series of target genes, such as pro-inflammatory factors, chemokines, and anti-apoptotic proteins, thus coordinating the initiation and amplification of inflammatory reactions (31–37). The non-canonical NF-κB signaling pathway is mainly involved in physiological activities, such as lymphatic organ development, B cell survival, and antibody production, which will not be explained in detail here.

#### 3.1.2 Amplification effects of alarmins

In addition to the above-mentioned inflammatory mediators, high mobility group protein 1 (HMGB1), S100 protein (S100A8 and S100A9), heat shock proteins (HSPs), and interleukin-33 (IL-33) are equally important in aseptic inflammation. They play a key role as alarm molecules or damage-associated molecular patterns (DAMPs; (15, 38)). In the frozen shoulder capsule, the level of HMGB1 increased significantly (10) and combined with Toll-like receptor 4 (TLR4) and receptor for advanced glycation end products (RAGE) on the surface of fibroblasts. The activation of the HMGB1-TLR4/RAGE axis further promotes NF-*κ*B to enter the nucleus, forming a vicious circle of inflammation and necrosis. More importantly, HMGB1 can also directly stimulate fibroblasts to secrete TGF-β1, thus establishing a molecular link between inflammation and fibrosis (15, 39, 40).

#### 3.1.3 Role of the NF-κB signaling pathway in frozen shoulder

Many studies have shown that the inflammatory reaction in the frozen shoulder is related to the NF-κB signaling pathway. In the tissue of frozen shoulders, the IL-17A produced by Th17 cells can cause fibroblasts to produce fibrosis and inflammatory reactions through the TRAF6/NF-κB pathway (41–43), and IKKβ inhibitors (NF-κB pathway inhibitors) can block this process (41). Further studies also studied the role of advanced glycation end products (AGEs) and their receptors (RAGE) in frozen shoulders. The results show that AGEs cause oxidative stress by binding to RAGE receptors and achieve this effect by activating the NF-κB pathway (15, 20, 44). Gurbuz and others also pointed out that melatonin can relieve joint capsule fibrosis by regulating oxidative stress and apoptosis through the NF-κB pathway (45).

### 3.2 Leptin/JAK–STAT signaling pathway

As a key regulatory factor for immunity and metabolism, the JAK–STAT signaling pathway is closely related to the inflammatory response (46–48). The disorder of this pathway, especially through the leptin resistance mechanism, can cause chronic low-degree inflammation (49). The systemic inflammatory environment caused by leptin resistance puts joint capsule fibroblasts in a pre-activated and sensitive state, causing them to overreact to mild mechanical damage or inflammatory stimulation and significantly reducing the threshold of fibrosis activation (4). Studies show that obese individuals usually show higher leptin levels than thin individuals, and the increase in leptin levels is closely related to frozen shoulders (especially for diabetics) (49, 50).

#### 3.2.1 Activation mechanisms of the JAK–STAT signaling pathway

Several cytokines (leptin, IL-2, IL-15) act on JAK–STAT signaling. Leptin is a polypeptide hormone secreted by fat, which is responsible for maintaining energy homeostasis (51). At the molecular level, leptin binds to the receptor, causing receptor dimerization and conformational changes, thereby activating the downstream Janus kinase 2 (JAK2). The tyrosine residue on the intracellular site of the activated JAK2 phosphorylated receptor is anchored to STAT3, and then, after recruitment and phosphorylation, STAT3 forms a homologous dimer (phosphorylated STAT3) and transfers to the nucleus (Figure 3) (52).

#### 3.2.2 Role of the JAK–STAT signaling pathway in frozen shoulder

After being transported to the nucleus, the dimerization of p-STAT3 has a dual function in the nucleus: on the one hand, it can bind to transcription factors such as NF-κB to enhance the transcription of pro-inflammatory cytokines and mediators, leading to an increase in local inflammatory reactions; on the other hand, it regulates participation in the cell cycle, gene expression for development, growth, and survival. This dual function of p-STAT3 serves as a molecular bridge between systemic metabolic disorders and the microenvironment of local inflammation of the shoulder joint (53). Further research found that there is a significant synergy between the pathological activation of the JAK–STAT pathway and the core pathway of fibrosis, the TGF-*β* signal. (54–56). In particular, the activation of STAT3 can directly upregulate the expression of TGF-β, and TGF-β can feed back to enhance the activity of STAT3, forming a mutually enhanced signal loop on the activation of fibroblasts, abnormal proliferation, and highly deposited ECM (type I and type III collagen), which leads to pathological thickening, contracture, and stiffness of the joint capsule. Therefore, the interaction between the JAK–STAT pathway and the TGF-*β* pathway is expected to be a key component of the continued development and slow progression of frozen shoulder fibrosis (4). It is worth noting that the disorder of the JAK–STAT pathway not only directly promotes fibrosis but also hinders the recovery of the disease by affecting the immune microenvironment. Perez et al. found that the defect of this pathway may disrupt the normal transformation of macrophages from the pro-inflammatory (M1) phenotype to the pro-repair (M2) phenotype (57). As a consequence, a pro-inflammatory microenvironment is retained, while initiated fibrosis does not evolve to an effective resolution, and thus the pro-immune and pro-fibrotic state are rarely co-existent (49). This relationship between immune–matrix interactions is considered to play a key role in the long frozen shoulder time and inability to resolve the inflammation fully.

### 3.3 Interconnectedness of inflammatory signaling pathways

NF-κB and leptin/JAK–STAT pathways also interact to amplify and maintain the inflammatory microenvironment of frozen shoulders. Activated STAT3 can interact with NF-κB p65 in the nucleus to enhance the transcription of pro-inflammatory cytokines (including IL-6 and IL-17A) and form and promote local inflammation (34, 53). In addition, NF-κB activates the expression of suppressor of cytokine signaling 3 (SOCS3), which participates in the JAK–STAT pathway, affecting the duration of the inflammatory stage (46). Importantly, these two pathways are jointly involved in the activation of TGF-*β* signals: STAT3 directly upregulates the transcription of TGF-*β*1, and the release of HMGB1 mediated by NF-κB promotes the secretion of TGF-β1 (40, 54).

## 4 Fibrotic formation phase: core TGF-*β* drive and imbalance in Wnt/β-catenin and hippo signaling pathways

Biological signs of fibrosis are abnormal activation of fibroblasts and excessive ECM deposition. It has been observed previously that TGF-*β*, WNT, and yes-associated protein 1 (YAP)/transcriptional coactivator with PDZ-binding motif (TAZ) signaling networks are key molecular drivers of fibrosis (58). TGF-β is a key fibrosis-promoting factor that upregulates excessive ECM synthesis genes through activating the nuclear transposition of the Smad complex (59). WNT promotes the activation of profibrotic target genes by stabilizing β-catenin and promoting its nuclear translocation (60). YAP is a mechanical signal sensor. YAP/TAZ responds to pathological matrix stiffness and induces fibroblasts to differentiate into myofibroblasts (58, 61).

While most of the studies focus on only one pathway, it has recently been suggested that the key elements of the pathways (Smad, *β*-catenin, and YAP/TAZ) intersect at the transcription level and at the protein level in a very complex regulatory network (58). The network cooperatively initiates fibrosis and is also able to maintain the current myofibroblasts active through a positive feedback loop, which provides a key molecular basis for the chronic progression of frozen shoulder and the persistence of joint contracture even after inflammation subsides.

### 4.1 TGF-*β* signaling pathway

The TGF-β signaling pathway is considered to be a classic driver of tissue fibrosis. As a chronic fibrosis disease of the shoulder capsule, frozen shoulder is closely related to this pathway (25, 62–71). TGF-*β* is highly elevated in frozen shoulder capsular tissue (72) and induces a variety of cellular fibrosis reactions, such as the production of ECM protein, fibroblast proliferation, increased differentiation of myofibroblasts, and collagen gel contraction (15).

#### 4.1.1 Mechanism of Smad-dependent TGF-β signaling pathway activation

TGF-β1 is believed to play a key role in shoulder joint capsule fibrosis (58, 70). The active TGF-β1 ligand binds to the type II receptor, which undergoes conformational change and phosphorylates the type I receptor to trigger its kinase activity (73, 74). Activated type I receptor phosphorylates cytoplasmic receptor-regulated Smads (Smad2/3), then phosphorylated Smad2/3 bind to the universal Smad4, which translocates to the nucleus to initiate transcription of fibrotic genes (Figure 3). The pathway operates through multi-step, tightly regulated events that drive fibrosis progression. Recently, miRNA has been shown to be particularly useful in fine-tuning this pathway (69, 75). In particular, miR-142 directly targets TGFBR2 mRNA to inhibit receptor production. *In vitro* experiments have shown that miR-142 overexpression blocks Smad-2/3 phosphorylation to suppress fibroticity (69), and miR-24 and miR-122 are found to be effective in regulating Smad signaling, which shows that the inactivation of the RNA interference mechanism is a crucial step in the pathological development (74).

#### 4.1.2 Role of the TGF-*β* signaling pathway in frozen shoulder

After the activation of the TGF-β1 pathway, the expression of its downstream target genes, such as encoding type I collagen, type III collagen, and *α*-smooth muscle actin (α-SMA), is significantly upregulated, leading to excessive collagen deposition in the ECM and tissue contracture, which together form the structural basis of joint stiffness (69). The evidence is that the pathway plays a critical role in human pathology. For example, in the secondary frozen shoulder case, a serum study revealed high expression of Smad3 and phosphorylated form in macrophages and fibroblasts, demonstrating activation in human pathology (76).

TGF-*β*/Smad is the core pathway of fibrosis, and studies have proven that inhibiting this signal pathway can alleviate fibrosis. For example, inhibiting Smad4 may inhibit the entire pathway, thus decreasing collagen deposition (70). A variety of natural compounds and proteins show inhibitory activity: tetrandrine blocks the pathway by inhibiting Smad2 activation (59, 71, 77); tumor necrosis factor-stimulated gene-6 (TSG-6) inhibits Smad2 phosphorylation (68, 78, 79); and intra-articular injection of relaxin-2 suppresses TGF-*β*1 expression by increasing cAMP levels (42). Nuclear receptor agonists such as PPAR-*γ* also reduce collagen deposition by inhibiting this pathway (62). All these make TGFβ/Smad a promising target.

Once this pathway is abnormally activated, it is closely related to the pathological environment of the frozen shoulder, and oxidative stress is one of the important upstream factors. In the joint capsule of patients with frozen shoulder, NOX4 is elevated, while antioxidant enzymes such as SOD1 and SOD2 is reduced, which leads to a continuous increase in the level of reactive oxygen species (ROS) (44, 80). High levels of ROS directly oxidize and activate the potential TGF-*β* and maintain a local chronic inflammatory state by aging fibroblasts and releasing senescence-associated secretory phenotype factors (81). Therefore, oxidative stress promotes the activation of TGF-*β*, and TGF-β will cause chronic inflammation and fibrosis, forming a vicious circle of self-circulation, closely connecting early inflammation and subsequent continuous fibrosis.

### 4.2 The Wnt/β-catenin signaling pathway

The Wnt/β-catenin pathway is an evolutionarily conserved developmental signaling pathway that plays a key role in apoptosis, tissue repair, and other processes. The study found that this pathway synergizes with the TGF-β pathway to promote the occurrence and development of fibrosis (82).

#### 4.2.1 Mechanisms of Wnt/β-catenin pathway activation

In the canonical Wnt pathway, Wnt ligands bind to Frizzled receptors and LRP5/6 auxiliary receptors on the cell membrane, thereby preventing β-catenin phosphorylation and ubiquitination. This will allow *β*-catenin to accumulate in the cytoplasm and then enter the nucleus to activate the target gene together with transcription factors of the TCF/LEF family. (Figure 3) (83).

#### 4.2.2 Role of the Wnt/β-catenin pathway in frozen shoulder

The Wnt signaling pathway is crucial during embryonic development, but it is abnormally activated in the frozen shoulder (60, 84). On the one hand, it independently promotes fibroblast activation, proliferation, and excessive ECM synthesis of local fibroblasts (58); on the other hand, it engages in extensive crosstalk with TGF-β and Hippo/YAP pathways to form a complex regulatory network to consolidate fibrotic phenotype (82, 85). Clinical research provides direct evidence: in the joint capsule tissue of patients with frozen shoulder, a significant increase in the level of nuclear β-catenin and an upward adjustment of its downstream target genes (such as insulin-like growth factor 2, IGF2) can be observed (86). In addition, the genome-wide association study has determined that the *WNT7B* site is an important risk site for frozen shoulders, and the core role of this pathway in the pathogenesis of the disease has been genetically established (2, 87).

### 4.3 Hippo signaling pathway

The Hippo signaling pathway is a highly conserved evolutionary pathway. Its core function is to regulate basic biological processes, such as organ size, cell proliferation, and apoptosis. It is closely related to the occurrence of cancer, tissue regeneration, and the maintenance of stem cell homeostasis (88–92). In recent years, more and more evidence shows that the components of this path and its cascade reactions play a key role in the occurrence and progression of fibrosis of multiple organs, such as the lungs, liver, kidneys, and heart (93–96). However, current research mainly focuses on these organs, and less attention is paid to the role of this pathway in joint tissue fibrosis. This research gap shows that studying the specific role and mechanism of the Hippo signaling pathway in joint soft tissue fibrosis, such as shoulder capsule freezing, is of great significance for a comprehensive understanding of disease pathology and finding potential intervention targets.

#### 4.3.1 Mechanisms of the hippo signaling pathway activation

The core function of the Hippo signaling pathway depends on the activity of its effector protein YAP/TAZ. This state is precisely regulated by the upstream kinase cascade reaction. When the Hippo pathway is activated, the upstream kinase cascade phosphorylates YAP/TAZ, enabling them to bind 14–3-3 proteins and remain cytoplasmic or undergo ubiquitin-mediated degradation, thereby completely inhibiting their transcriptional co-activator function. On the contrary, when this pathway is inhibited (for example, affected by cytoskeleton tension signals), the upstream kinase will be inactivated. YAP/TAZ maintains a dephosphorylated state and can enter the nucleus and combine with transcription factors such as the TEA DNA-binding domain to form an active complex. This will activate the expression of target genes and promote cell proliferation, migration, and synthesis of ECM (58, 61, 97–101).

#### 4.3.2 Role of the hippo/YAP pathway in frozen shoulder

Although there is no direct research on the specific role of the Hippo/YAP signaling pathway in frozen shoulder, the study of the mechanism of this pathway in various organ fibrosis diseases provides important clues for understanding the pathogenesis of this disease. As the core effector of this pathway, YAP/TAZ has been proven to be the key to cell perception and response to mechanical signals. In the process of fibrosis, the increase in the stiffness of the ECM will directly promote the transfer of YAP/TAZ into the nucleus, thus activating the differentiation of fibroblasts into myofibroblasts, leading to excessive ECM deposition (94, 102). From this, we speculate that in the pathological environment of frozen shoulder, the stiffened and contracted shoulder capsule generates aberrant mechanical stress. This mechanical environment will activate YAP/TAZ, thus promoting local fibroblast activation and ECM metabolic imbalance, which may be the key reason for the long-term existence of frozen shoulder. Research shows that YAP/TAZ can interact with the components of TGF-*β*/Smad and Wnt/β-catenin signaling pathways to form transcription complexes and jointly enhance the expression of fibrosis-promoting genes (61).

### 4.4 Interconnectedness of fibrotic signaling pathways

During fibrosis, there is also interaction among the TGF-β, Wnt/β-catenin, and Hippo/YAP pathways. The TGF-β/Smad signaling pathway induces Wnt ligand expression and stabilizes β-catenin, enhancing Wnt/β-catenin activity (58). In addition, nuclear β-catenin can form a complex with Smad3 to jointly regulate the target genes associated with fibrosis and promote the transcription effect (85). The Hippo effector YAP/TAZ, as the central mechanical transduction node, integrates inputs from TGF-β and Wnt signals. TGF-β-induced cytoskeletal remodeling promotes YAP/TAZ nuclear transposition, and stable β-catenin interacts with TAZ to enhance its transcriptional activity (94, 95). In the process, the gradual hardening of the ECM further strengthens the nuclear localization of YAP/TAZ (102).

## 5 The PI3K-Akt signaling pathway

The PI3K-Akt signaling pathway plays a role in regulating the growth, proliferation, survival, and metabolism of cells. Studies show that this pathway also plays an important role in fibrosis, especially in the activation and migration of fibroblasts (103, 104).

### 5.1 Mechanisms of PI3K-Akt pathway activation

The activation of the PI3K-Akt signaling pathway is triggered by the combination of extracellular growth factor and its corresponding receptor tyrosine kinase, triggering receptor dimerization and self-phosphorylation. The phosphorylated tyrosine site recruits PI3K into the cell membrane through the SH2 domain. The activated PI3K converts phosphatidylinositol (4,5)-bisphosphate (PIP2) into the second messenger phosphatidylinositol (3,4,5)-trisphosphate (PIP3), and further recruits Akt and PDK1 containing PH domains to the membrane region. The activated Akt then phosphorylates downstream target proteins to regulate related processes, including cell survival, proliferation, and metabolism. The pathway is strictly negatively regulated by PTEN, and PTEN terminates signal transduction by dephosphorylating PIP3 into PIP2 (105–107).

### 5.2 Role of the PI3K-Akt signaling pathway in frozen shoulder

There is evidence that the PI3K-Akt pathway is a key contributor to changing shoulder capsule fibrosis, which directly contributes to disease progression by promoting abnormal activation, growth, and survival of synovial fibroblasts (3, 98–101). There are many pathological factors upstream of this pathway, including the high expression of IL-6 in fibroblast patients that promotes fibrosis by activating the PI3K-Akt pathway. If siRNA inhibits IL-6, the phosphorylation level of Akt will decrease, attenuating the degree of fibrosis (3). In addition, CD36 is also highly expressed in frozen shoulder, which aggravates fibrosis with activation of the pathway. Yan et al. noted that Salvianolic acid-B (SBA-B) can effectively block signals by inhibiting CD36, thus reducing inflammation and fibrosis (101). Endogenous hormones are also involved in regulating this pathway. Wang et al. found that estrogen inhibits the phosphorylation of PI3K and Akt through its receptor GPER, thus preventing the activation of fibroblasts and reversing the fibrosis process (99). This mechanism provides molecular biological significance for the high number of perimenopausal women undergoing periarthritis. Physical treatment can also control this pathway. Animal studies show that tuina therapy can regulate the protein expression of the PI3K-Akt pathway in the capsule of rats with periarthritis, reverse the pathological changes in fibrosis, and thereby improve the mobility of the shoulder joint (98).

## 6 The HIF-1 pathway

HIF-1α is a regulatory factor in the progression of fibrosis. Its mechanism involves multiple pathways, such as cell cycle regulation, epithelial–mesenchymal transition, and signal transduction crossover.

### 6.1 Mechanisms of the HIF-1 pathway activation

The HIF-1 signaling pathway is the key mechanism for cells to adapt to a hypoxic environment. It maintains the balance of cells and tissues by sensing changes in oxygen concentration and responding to them. The pathway is mainly composed of heterologous dimer transcription factors, which contain HIF-1α and HIF-1*β* subunits, of which HIF-1α is an oxygen-dependent regulatory subunit and HIF-1β is a continuous expression subunit. Under normal oxygen conditions, the proline residue (Pro-402/Pro-564) of HIF-1α will be hydroxylated by prolyl hydroxylase (PHD1-3), thus triggering binding to the von Hippel–Lindau (VHL) protein. Subsequently, it will lead to its degradation through the ubiquitin–proteasome pathway. Under hypoxic conditions, PHD activity will be inhibited, so HIF-1α will accumulate steadily and transfer to the nucleus. There, it binds to HIF-1β to form a complete transcription factor and activates the hypoxic response element in the target gene promoter region to regulate the expression of downstream genes (108, 109).

### 6.2 Role of the HIF-1 pathway in frozen shoulder

**Figure 4:**
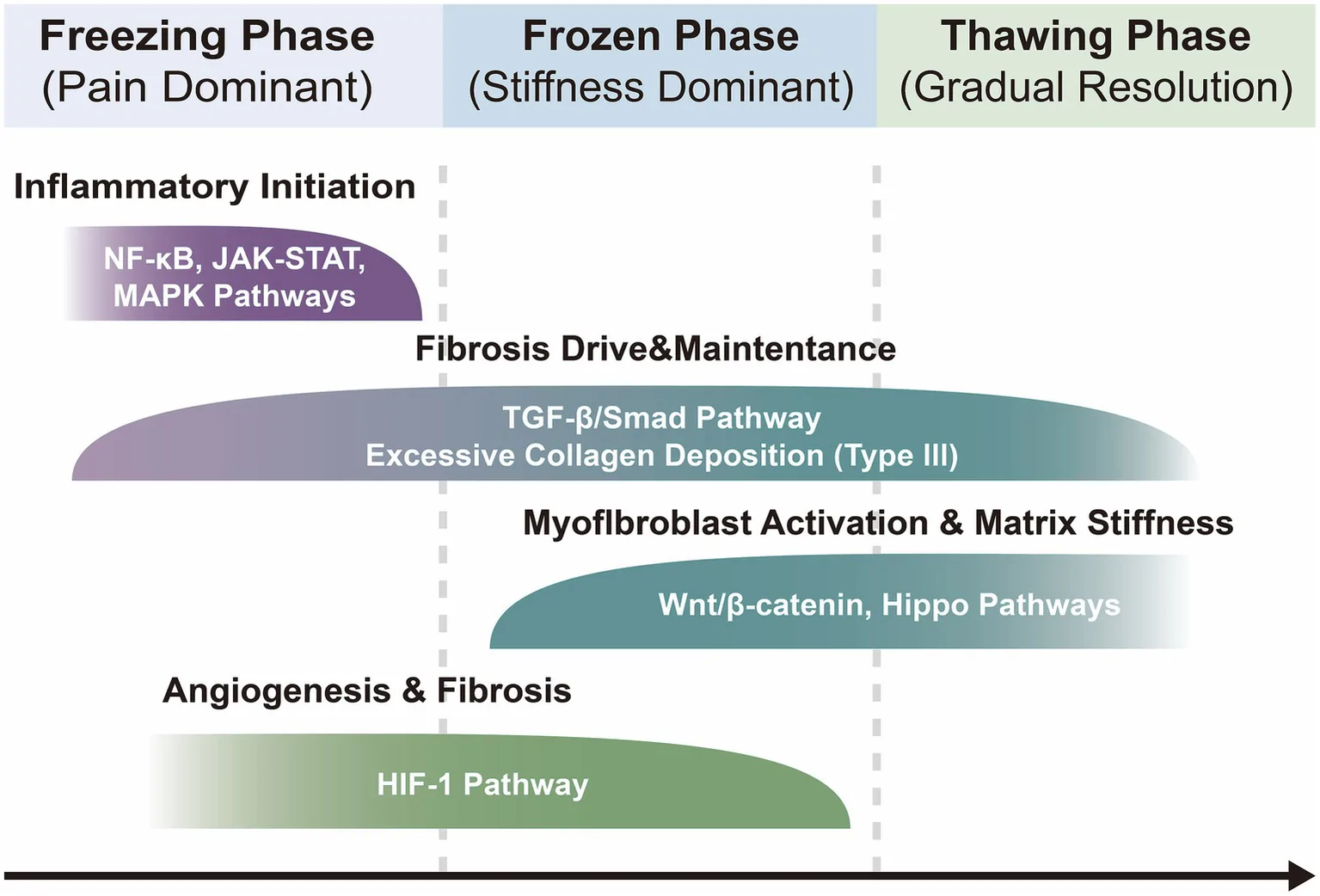
An integrated schematic of the clinical–pathological–molecular progression in adhesive capsulitis. In the freezing phase, NF-κB and leptin/JAK–STAT pathways are predominantly activated by alarmins and pro-inflammatory cytokines, initiating synovitis. During the frozen phase, TGF-β/Smad, Wnt/β-catenin, and Hippo/YAP become dominant, driving fibroblast activation, myofibroblast differentiation, and excessive ECM deposition. PI3K-Akt and p38 MAPK sustain fibroblast survival, while HIF-1α responds to hypoxia and promotes angiogenesis. In the thawing phase, inflammation subsides, but fibrosis resolves slowly due to persistent YAP/TAZ mechanotransduction.

In frozen shoulder, contracture and joint capsule will lead to insufficient blood supply to the tissue, hypoxia, and then lead to redox imbalance. These pathological processes will activate HIF-1α. Early arthroscopy shows synovial congestion and capillary network proliferation (8), which is a manifestation of HIF-1α activation. This pathological change is related to the expression of VEGF induced by HIF -1α and pathological angiogenesis (109–114). In addition to promoting angiogenesis, HIF-1α also promotes fibrosis through a variety of mechanisms, leading to the chronic progression of the disease. First of all, it directly upregulates collagen cross-linking enzymes, thus increasing collagen deposition and tissue hardness. Second, HIF-1α forms a positive feedback loop with the TGF-*β*/Smad signaling pathway. It may further aggravate fibrosis and tissue contracture by inducing epithelial–interstitial transformation and enhancing fibroblast activity (Figure 4). The latest study also shows that the HIF-1 pathway may interact with the process of iron death and participate in the pathological regulation of frozen shoulder. In frozen shoulder tissue, genes associated with iron death showed differences, most of which were related to the HIF-1 signaling pathway and inflammatory reactions (115–121).

## 7 Other relevant signaling pathways and mechanisms

### 7.1 The MAPK signaling pathway

In the pathological process of frozen shoulder, the signaling pathway of mitogen-activated protein kinase (MAPK), especially the p38 MAPK sub-pathway, acts as a key molecular hub to regulate the progression of inflammatory response and fibrosis (98, 122, 123). This pathway not only directly regulates the proliferation, migration, and collagen synthesis of fibroblasts, but studies also show that it may be a key mechanism bridging joint capsule fibrosis of the frozen shoulder and the accompanying osteoporosis (122, 124).

In a basic study, TAK715 (a specific p38 MAPK inhibitor) can effectively reverse the fibrotic phenotype of synovial fibroblasts, while improving the ROM and osteoporosis in animal models (122). Further research shows that the new antioxidant RuCo nanosheet can significantly inhibit the fibroblastic activity of fibroblasts by inhibiting the p38 MAPK signal, thus alleviating shoulder stiffness and joint capsule fibrosis in mouse models (125). In addition, clinical-related physical intervention studies also provide evidence of the importance of this pathway: a phosphoproteinomic analysis found that differentially phosphorylated proteins were significantly enriched in the MAPK pathway after tuina treatment, which suggests that the regulation of the pathway may be one of the mechanisms of tuina treatment (98).

### 7.2 Hedgehog and notch signaling pathways

Large-scale whole-genome association studies revealed that Dupuytren’s disease is also associated with frozen shoulder, and that Hedgehog and Notch signaling pathways might play an important role in fibrosis, providing a new insight into what is common in frozen shoulder and other fibrotic diseases (126).

### 7.3 Genetic susceptibility factors

Case–control studies have identified specific genetic variants in ECM homeostasis-related genes (such as *MMP13, MMP9, TGFB1, and TGFBR1*) as being associated with susceptibility to frozen shoulder (10, 63, 64, 66, 127–130). Variants in ECM homeostasis-related genes may influence how tissues respond to injury, altering the kinetics and severity of the fibrotic process (128).

## 8 New perspectives from single-cell transcriptomics

Fibroblasts exist in different subsets and play different roles in tissue homeostasis, inflammation, and fibrosis (131–134). Single-cell sequencing studies have shown that DKK3 + and POSTN+ fibroblasts, a subset with high Dickkopf-3 and Periostin expression, have transcriptional profiles highly similar to those of cells during fetal shoulder development, suggesting that the pathological process of frozen shoulder may involve abnormal reactivation of embryonic developmental programs (135).

## 9 Conclusions and prospects

The process of frozen shoulder fibrosis is a complex pathological network involving the interweaving of multidimensional pathways. Existing research has made progress in pathological signaling pathways, but the key limitations include that the interaction network of core signaling pathways has not been fully clarified. It has been confirmed that systemic factors, such as diabetes, obesity, and thyroid abnormalities, are high-risk factors for frozen shoulder, but the specific molecular chain of systemic metabolic/inflammatory abnormalities (such as leptin resistance and AGEs accumulation) to downshift the threshold of shoulder capsule fibroblast repair and connect and activate local pathways is not clear (1, 15). There is complex crosstalk between PI3K-Akt, TGF-*β*/Smad, MAPK, and NF-κB pathways, which are driven at different pathological stages. The molecular mechanism of mutual regulation and collaboration of fibrosis is still unclear. For example, although the crosstalk between TGF-β, Wnt/β-catenin, and YAP/TAZ has been well recorded in organ fibrosis, there is still little direct evidence from the human frozen shoulder capsule; most inferences are derived from other tissues or *in vitro* fibroblast culture (58). This hinders the systematic understanding of the whole pathological network of frozen shoulder. At the same time, the upstream triggers and the activation mechanism of the downstream pathway need to be further clarified, and the influence of genetic background on pathway activity also needs to be further explored. Breaking through these bottlenecks helps to establish a more complete pathological map and lays the foundation for personalized treatment based on pathway regulation. Furthermore, existing targeted interventions (such as TGF-β/Smad inhibitors, Tetrandrine) mostly stay in the in vitro cell or conventional animal model stage and lack the efficacy verification of the corresponding human pathological staging. Personalized treatment strategies have not been established in combination with pathway activity and genetic background, and the molecular regulation mechanism of clinical routine treatment does not fully match the existing pathway research conclusions.

Future research should systematically study the joint capsule samples of patients at different stages through multi-omics integration analysis; combine proteomics, phosphorylated proteomics, transcriptomics, and metabolomics techniques; draw a signal pathway network map; and reveal the interaction mechanism of key pathways. In-depth analysis of the signal pathway of frozen shoulder fibrosis helps to find out the cause and lays a theoretical foundation for precise treatment. The combination of basic research and clinical application is expected to provide more effective treatment methods and significantly improve the quality of life of patients.
